# Patients’ related sexual outcomes in colorectal surgery

**DOI:** 10.3389/fonc.2022.968978

**Published:** 2022-12-16

**Authors:** Emilie Liot, Niki Christou, Sandra de Sousa, Jacques Klein, Iranmanesh Pouya, Danae Guedj, Nicolas C. Buchs, Frédéric Ris

**Affiliations:** ^1^ Division of Digestive Surgery, University Hospitals of Geneva, Genève, Switzerland; ^2^ Unit of Surgical Research, University of Geneva, Genève, Switzerland; ^3^ Service de Chirurgie Digestive, Endocrinienne et Générale, University Hospital of Limoges (CHU) de Limoges, Limoges, France; ^4^ Division of Urologic Surgery, Department of Surgery, University Hospitals, Genève, Switzerland

**Keywords:** sexual function, marital satisfaction, colorectal surgery, assessment, patient related outcome

## Abstract

**Background:**

Patients undergoing colorectal surgery (CRS) have an increased risk of developing sexual disorders, attributed to different mechanisms. In this context, sexual function (SF) assessment of patients before and after surgery is essential: to identify risk factors for sexual disorders as well as to minimize their impact on overall quality of life (QoL), allowing them a satisfying relationship and sexual life.

**Material and methods:**

Patients over 18 years of age who underwent a CRS in the University Hospital of Geneva, Switzerland, between June 2014 and February 2016 were included. Our main objective was to compare and analyze the evolution of SF, QoL, and marital satisfaction (MS) before and after CRS. Specific and standardized tests were used.

**Results:**

A cohort of 72 patients with a median age of 58.73 was analyzed. The majority of CRS was elective (91.5%). A percentage of 52.8% of patients underwent surgery for oncological reasons. There was no statistical difference in SF, sexual QoL, and MS before and after elective or emergency CRS for men. Interestingly, a significant decrease in women’s SF (FSFI) as well as their satisfaction within their couple (Locke–Wallace) until 12 months after surgery was found (p = 0.021). However, they showed a steady SF (GRISS) within their couple until 12 months after surgery.

**Conclusion:**

Regarding knowledge about difficulties to talk about this intimate topic and gender differences, this general overview raises the question of the necessity to introduce in a long-course follow-up different methods of sexual health assessment with specific stakeholders.

## Introduction

Colic and rectal resections are common procedures performed daily within a department of general surgery. It encompasses many benign and malign pathologies. The majority of publications linked to colorectal surgery aim at highlighting different kinds of outcome such as mortality, morbidity, and oncological/disease results.

However, with the improvement in the management of colorectal pathologies due to minimally invasive techniques (laparoscopy, robot-assisted surgery) (1), the adjunction of therapies like chemotherapy and/or radiotherapy in case of cancer (2), but also with the increase of inflammatory bowel disease (IBD) diagnosis especially among young patients (3, 4), it is necessary to assess specific outcomes after colorectal surgery. Thus, functional results and potential complications within the domain of sexuality have to be evaluated. Sexual function is one of the aspects of quality of life that may be disrupted after surgery. In case of colorectal surgery, sexual disorders appear to be multifactorial. During dissection, the superior and/or inferior hypogastric plexus may be damaged and linked to major sexual disorders like erectile dysfunction, problems of ejaculation, decrease of libido or lubrification, and dyspareunia (5). Psychological stress and body image modifications due to the surgery also imply sexuality alterations (6). Finally, the type of colorectal pathology with its dissemination/extension in the pelvis (colorectal cancers, inflammatory bowel diseases) and also its specific medical treatments can modulate sexual functions (7).

The aim of this study is to assess the impact of colorectal surgery on both sexual function and quality of life of patients and their partners.

## Materials and methods

### Data source

This monocentric prospective study focused on patients who underwent a colorectal surgery between June 2014 and February 2016 at Geneva University Hospitals, Switzerland, and was approved by the Health Research Ethics Board at the University of Geneva (CER 14-111).

### Patient population

Inclusion criteria were heterosexual patients in a stable relationship understanding French and having benefited from elective or emergency colorectal surgery in Geneva University Hospital for colorectal cancer or diverticular or bowel inflammatory diseases. No patient was included in the database twice.

Pregnant patients, patients under 18 years of age, those who have fulfilled only one questionnaire, those who died during the study, patients without sexual activity, patients with tumoral progression during the follow-up or having left the study, and homosexual patients were excluded.

### Methods

This study compared sexual function and marital satisfaction before and after colorectal surgery in both men and women.

All participating subjects provided written informed consent.

### Questionnaires

Validated questionnaires were given to patients waiting for elective or emergency colorectal surgery before (before surgery or during the hospital stay according to the degree of emergency) and after (at 3, 6, and 12 months follow-up) surgery. They were filled out without help.

For the assessment of the sexual function, gender-specific questionnaires were used: the International Index of Erectile Function for men [IIEF] and the Female Sexual Function Index for women [FSFI].

For the evaluation of the quality of life, the Locke–Wallace relationship adjustment test [marital satisfaction] and the GRISS [quality of sexual life] were used.

The IIEF is a 15-item questionnaire, assessing all dimensions of male sexual function: erectile function, orgasmic function, sexual desire, intercourse satisfaction, and overall satisfaction (8). Each item is scored on a five-point scale, and the overall score (OS) (minimum: 5 to maximum: 75 points) is obtained by adding each item score. Erectile dysfunction (ED) is classified as severe ED (OS between 1 and 10), mild to moderate ED (11–25), and no ED (≥26).

The FSFI questionnaire is a 19-item questionnaire, assessing all aspects of female sexual function: desire, arousal, lubrication, orgasm, satisfaction, and pain (9). Each item is scored on a six-point scale with an OS (minimum of 2 and maximum of 36 points) obtained by combining each item score. An OS lower than 23 defines a poor sexual function, an OS between 23 and 29 means a good sexual function, and an OS greater than 29 corresponds to a very good sexual function. The overall score is lower than 26 in the presence of one or more dysfunctions in specific areas.

The Locke–Wallace Marital Adjustment Test is a 15-item test, assessing the level of couple satisfaction by underlining the extension of agreement/disagreement between partners. Each item is scored from 0 to 35. OS (a minimum 2 and a maximum of 158 points) is obtained by adding each item score. The severity of issues encountered by the partners can be classified as serious (OS <80), difficulties (OS 80–100), and no problems (OS >100) (10).

The GRISS is a 28-item questionnaire assessing the existence and severity of sexual problems within the couple and for each partner. There is a version for each gender. Various aspects of the relationship are explored: communication, non-genital physical contact, dissatisfaction, avoidance of sexual intercourses, frequency of sexual activity, and impotence and premature ejaculation for men and anorgasmia and vaginismus for women. The obtained overall score measures the sexual dysfunction: the higher the score, the greater the sexual dysfunction. The score ranges from 0 to 10, and values higher than 5 indicate sexual dysfunction.

### Outcomes and covariates definition

The main outcome was the evolution of both sexual function and marital satisfaction of patients after colorectal surgery.

### Statistical analysis

Results are expressed as medians with interquartile range (IQR) for quantitative variables and qualitative variables.

Comparisons before and after surgery were done with ANOVA test (analysis of variance).

In order to bring out a significant change concerning the different tests before and after surgery, inclusion of a minimum of 20 patients in each group was necessary. Around 200 patients are operated each year for a colorectal pathology in the department, and considering a minimum attendance, a satisfactory statistical power could be achieved in 2 years.

## Results

**Figure 1:**
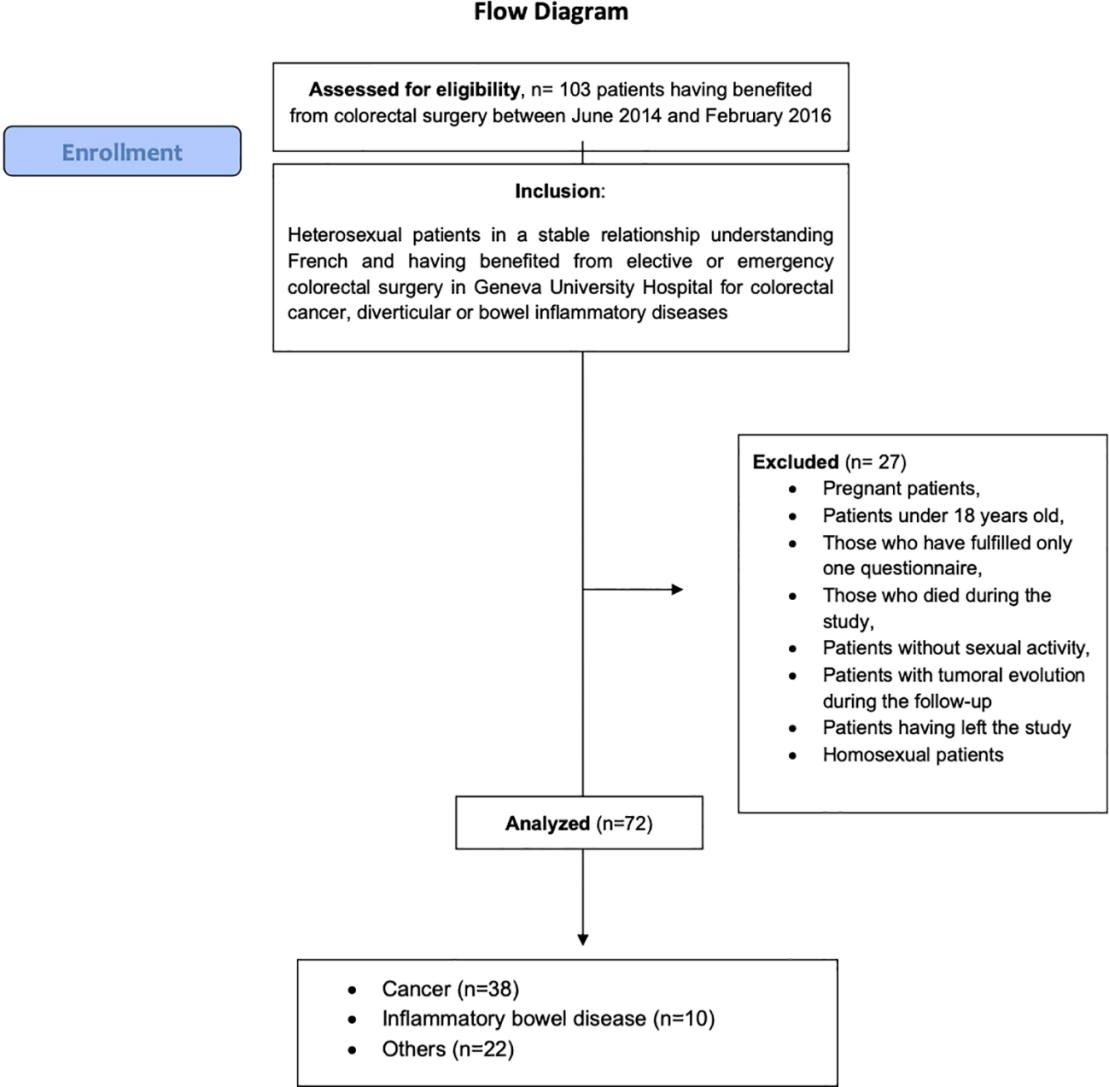
Flow diagram.

Questionnaires were proposed to 103 patients. Of these patients, 31 did not meet the inclusion criteria. After the exclusion process, 72 patients were included (**Figure 1**).

Among the 72 patients, 56 (77.8%) were men and 16 (22.2%) were women. Patients’ characteristics are described in **Table 1** with a median age of 58.73 years (50.37–68.54). The median body mass index (BMI) was 25.60 kg/m² (23.58–28.40).

**Table 1:** Characteristics of the population included.

	Female (N = 16)	Male (N = 56)	Total (N = 72)
Age			
Median (Q1, Q3)	56.59 (48.15, 68.67)	58.73 (50.65, 68.48)	58.73 (50.37, 68.54)
Min–max	36.98–73.37	25.69–83.89	25.69–83.89
Indication			
Adenoma (low-grade dysplasia)	0 (0.0%)	1 (1.8%)	1 (1.4%)
Inflammatory bowel disease	1 (6.2%)	9 (16.1%)	10 (13.9%)
Cancer	9 (56.2%)	29 (51.8%)	38 (52.8%)
Non-inflammatory bowel disease	6 (37.5%)	16 (28.6%)	22 (30.6%)
Polyp (high-grade dysplasia)	0 (0.0%)	1 (1.8%)	1 (1.4%)
Localization			
Unique			
Right colon	1 (6.2%)	7 (12.5%)	8 (11.1%)
Transverse colon	0 (0.0%)	1 (1.8%)	1 (1.4%)
Left colon	2 (12.5%)	3 (5.4%)	5 (6.9%)
Sigmoid	8 (50.0%)	27 (48.2%)	35 (48.6%)
Rectum	4 (25.0%)	13 (23.2%)	17 (23.6%)
Double			
Colon and rectum	0 (0.0%)	1 (1.8%)	1 (1.4%)
Ileum and cecum	1 (6.2%)	3 (5.4%)	4 (5.6%)
Sigmoid and appendix	0 (0.0%)	1 (1.8%)	1 (1.4%)
Neoadjuvant radiochemotherapy			
No	8 (66.7%)	38 (74.5%)	46 (73.0%)
Yes	4 (33.3%)	13 (25.5%)	17 (27.0%)
Emergency			
No	16 (100.0%)	49 (89.1%)	65 (91.5%)
Yes	0 (0.0%)	6 (10.9%)	6 (8.5%)
Diabetes			
No	16 (100.0%)	52 (94.5%)	68 (95.8%)
Yes	0 (0.0%)	3 (5.5%)	3 (4.2%)
Smoking			
No	9 (56.2%)	31 (57.4%)	40 (57.1%)
Yes	7 (43.8%)	23 (42.6%)	30 (42.9%)
Immunodepression			
No	14 (87.5%)	47 (88.7%)	61 (88.4%)
Yes	2 (12.5%)	6 (11.3%)	8 (11.6%)
BMI			
Median (Q1, Q3)	25.85 (22.67, 28.63)	25.45 (23.60, 28.40)	25.60 (23.58, 28.40)
Min–max	19.00–36.10	19.80–33.50	19.00–36.10
Psychiatric_disorder			
No	13 (81.2%)	50 (90.9%)	63 (88.7%)
Yes	3 (18.8%)	5 (9.1%)	8 (11.3%)
Dyslipidemia			
No	12 (75.0%)	46 (83.6%)	58 (81.7%)
Yes	4 (25.0%)	9 (16.4%)	13 (18.3%)
Alcohol			
No	15 (93.8%)	37 (67.3%)	52 (73.2%)
Yes	1 (6.2%)	18 (32.7%)	19 (26.8%)
Cardiovascular pathology			
No	13 (81.2%)	36 (65.5%)	49 (69.0%)
Yes	3 (18.8%)	19 (34.5%)	22 (31.0%)

Various comorbidities were present within the cohort: active smoking (42.9%), cardiovascular background (31.0%), regular alcohol consumption (26.8%), dyslipidemia (18.3%), immune insufficiency (11.6%, due to immunosuppressive therapy such corticosteroids), psychiatric disorders (11.3%), and diabetes (4.2%).

Most surgical interventions (91.5%) were elective procedures. In 52.8% of cases, surgery was performed for oncological reasons and in 30.6% for benign non-inflammatory bowel diseases. The disease had double localization (small and large intestine) in 8.4% of cases whereas it was only localized in the colon in 19.4%, in the rectum in 23.6%, or in the sigmoid in 48.6%. There were 17 of 38 oncological patients (44.7%) who received a neoadjuvant treatment.

### Preoperative setting

At the time of surgery, women had a mean FSFI overall score of 28 indicating a good sexual function, a mean overall score for the Locke–Wallace Marital Adjustment Test of 118 meaning a good agreement within the couple, and a mean GRISS score of 61 (**Tables 2**, **3**).

**Table 2:** Overall results for marital satisfaction and sexual quality of life.

	Preoperative period (N = 72)	3 months after surgery (N = 72)	6 months after surgery (N = 72)	12 months after surgery (N = 72)	Total (N = 288)
**GRISS total**					
Median (Q1, Q3)	64.0 (58.5, 66.5)	62.0 (59.5, 66.0)	63.0 (59.7, 67.0)	61.0 (57.5, 65.0)	63.0 (58.0, 66.0)
Min–max	45.0–93.0	44.0–85.0	53.0–73.0	53.0–76.0	44.0–93.0
**Locke–Wallace**					
Median (Q1, Q3)	124.5 (103.2, 139.0)	118.0 (100.5, 135.7)	120.5 (105.2, 132.7)	121.5 (97.0, 131.7)	121.5 (101.2, 136.0)
Min–max	12.0–156.0	14.0–157.0	34.0–148.0	37.0–151.0	12.0–157.0

**Table 3:** Summary outcomes for women.

	preop (N = 16)	3 months (N = 16)	6 months (N = 16)	12 months (N = 16)	Total (N = 64)
GRISS_total					
Mean (SD)	65.14 (9.27)	62.50 (5.80)	67.82 (4.38)	65.36 (6.12)	65.26 (6.89)
Median (Q1, Q3)	63.50 (61.00, 67.00)	63.00 (60.00, 66.25)	68.00 (63.50, 71.50)	63.00 (61.50, 69.50)	64.00 (61.25, 69.75)
Min–max	53.00–93.00	50.00–70.00	62.00–73.00	57.00–76.00	50.00–93.00
Locke–Wallace					
Mean (SD)	119.53 (24.20)	124.90 (18.16)	104.70 (33.96)	107.82 (30.45)	114.67 (27.38)
Median (Q1, Q3)	126.00 (104.00, 137.50)	128.50 (113.75, 135.75)	119.50 (83.00, 126.50)	121.00 (84.50, 130.50)	124.50 (102.25, 134.75)
Min–max	63.00–152.00	92.00–152.00	34.00–140.00	52.00–141.00	34.00–152.00
IFSF_total					
Mean (SD)	28.09 (5.40)	24.97 (9.51)	19.18 (12.52)	22.68 (8.52)	24.15 (9.32)
Median (Q1, Q3)	30.00 (26.25, 31.75)	30.00 (22.70, 30.90)	20.60 (7.45, 30.60)	23.25 (19.23, 28.52)	27.30 (19.80, 31.17)
Min–max	14.10–35.40	2.90–33.00	1.90–34.80	2.30–35.00	1.90–35.40

For men, the preoperative mean IIEF score was 49 corresponding to no erectile dysfunction, the mean overall score for the Locke–Wallace Martial Adjustment Test was 119 showing no disagreement between partners, and the mean GRISS score was 62 (**Tables 2**, **4**).

**Table 4:** Summary outcomes for men.

	preop (N = 56)	3 months (N = 56)	6 months (N = 56)	12 months (N = 56)	Total (N = 224)
GRISS total					
Mean (SD)	61.67 (6.37)	61.24 (8.11)	61.31 (4.69)	60.53 (4.27)	61.23 (6.08)
Median (Q1, Q3)	64.00 (56.00, 66.00)	62.00 (58.00, 66.00)	61.00 (57.00, 65.00)	60.00 (57.00, 63.50)	62.00 (57.00, 65.00)
Min–max	45.00–73.00	44.00–85.00	53.00–70.00	53.00–70.00	44.00–85.00
Locke–Wallace					
Mean (SD)	118.96 (26.54)	111.75 (31.35)	119.22 (20.80)	113.95 (27.61)	116.09 (26.89)
Median (Q1, Q3)	122.00 (103.50, 141.50)	111.00 (98.50, 132.50)	120.50 (109.75, 137.50)	122.00 (98.00, 131.00)	120.00 (100.75, 137.00)
Min–max	12.00–156.00	14.00–157.00	80.00–148.00	37.00–151.00	12.00–157.00
IIEF total					
Mean (SD)	48.84 (19.84)	46.59 (18.55)	49.32 (18.70)	48.49 (21.50)	48.26 (19.57)
Median (Q1, Q3)	51.50 (41.50, 65.00)	48.00 (35.00, 59.75)	56.00 (38.00, 62.25)	53.00 (35.00, 67.00)	52.00 (36.50, 65.00)
Min–max	7.00–74.00	5.00–71.00	5.00–74.00	5.00–75.00	5.00–75.00

### Postoperative setting and evolution

**Figure 2:**
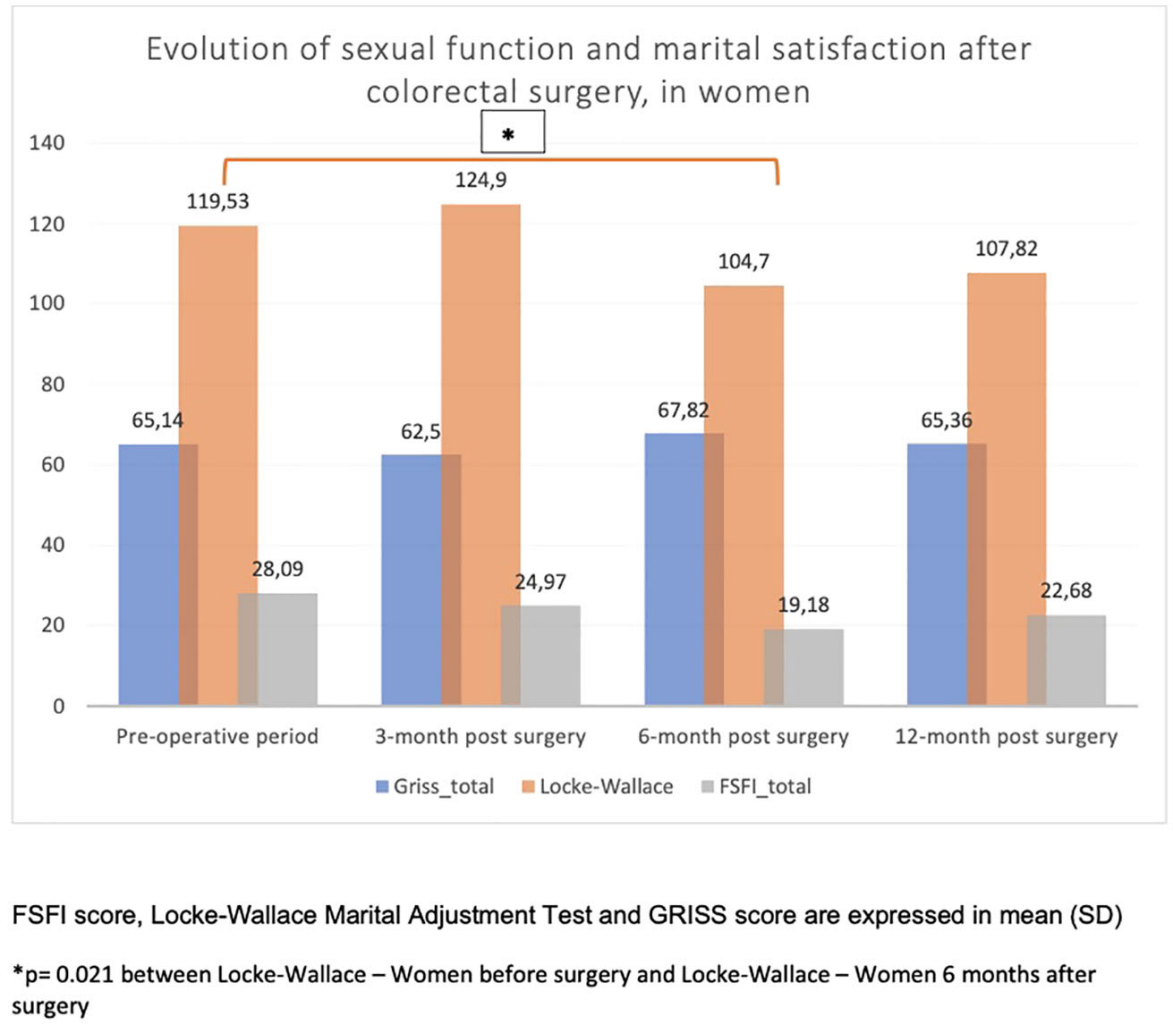
Evolution of sexual function and marital satisfaction after colorectal surgery, in women.

After surgery, women indicated that their own sexual function (FSFI) slightly decreased until 12 months (**Figure 2**) as well as their satisfaction within their couple (Locke–Wallace Marital Adjustment Test) (p = 0.021 between LWAT scores before surgery and 6 months after surgery) (**Figure 2** **).** However, they showed a steady sexual function (GRISS) within their couple until 12 months after surgery (**Figure 2**).

**Figure 3:**
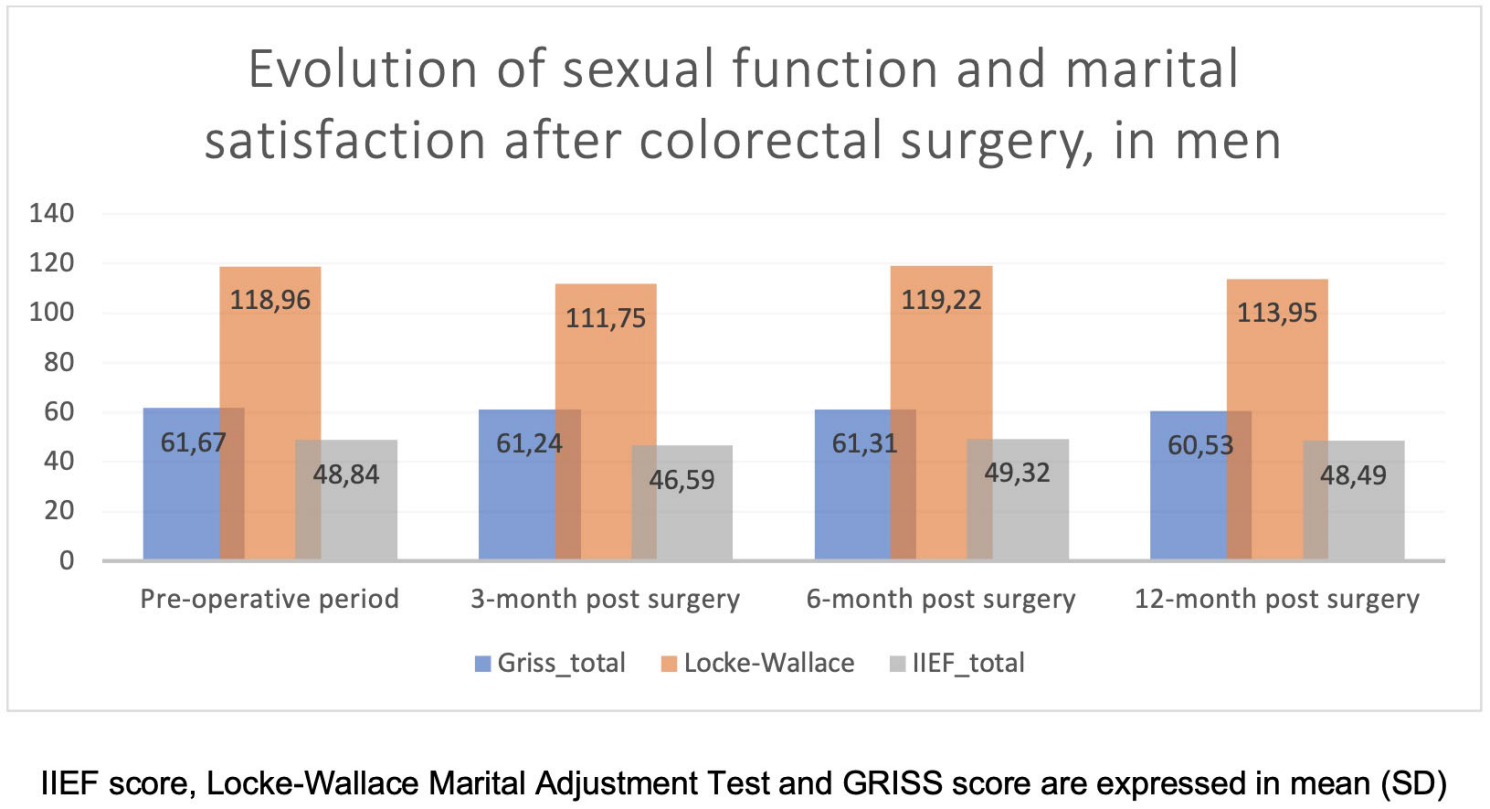
Evolution of sexual function and marital satisfaction after colorectal surgery, in men.

Regarding men, they assessed their own sexual function as quite stable (IIEF) until 12 months after surgery as well as their satisfaction within their couple (Locke–Wallace) and their sexual function within their couple (GRISS) (**Figure 3**).

## Discussion

One of the interests of this study is to highlight sexuality, a subject too often taboo for caregivers but nevertheless essential for the quality of life of their patients. Thanks to this research, we have a detailed view of the sexual health of the patients as well as their development within their couple before surgery. Interestingly, we find that patients waiting for elective surgery have an overall satisfactory sexuality and relationship.

Moreover, this study demonstrates that colorectal surgery regardless of indication (including inflammatory disease and oncological pathology) and location of the intestinal resection (right colon, transverse colon, sigmoid, rectum, or both) is not linked to modifications of sexual functions, sexual quality of life, or marital satisfaction.

After reviewing the literature, most studies in the field of colorectal surgery analyze mortality and morbidity and aim to identify their risk factors. However, few of them are interested in functional complications of such procedures like sexual dysfunctions. These are focused on oncological surgery and especially in the subgroup of men (11) and the rectal location (12–14).

Indeed, in colorectal surgery, the surgical procedures of the rectum are more concerned about sexual dysfunctions as pelvic localization means dissection along the superior and/or inferior hypogastric plexus contributing to the innervation of genital organs. Moreover, men are more concerned by colorectal cancer: around 1 million of men versus 846,000 women in 2020 worldwide (15) despite a trend to women in some countries (16).

Following this statement, at first sight, one limit of our work is to have encompassed both localization of the colon and rectum. However, it is important here to stress out that colon surgery in the right or transverse colon, which are not localized into the pelvis, and regardless of indication for surgery, may also lead to sexual dysfunctions. Indeed, sexual function is linked to several factors. Among them, we can point out psychological factors (stress, apparition of a disease, modifications of the body image with presence of potential stoma bag, issues within relationships, patients’ social situation, etc.) and physical issues due to treatments (medications, chemotherapy, biotherapy, radiotherapy, surgery). Consequently, not only rectal surgery with its specific localization but also colonic surgery should be given special attention regarding sexual dysfunctions through a biopsychomedical perspective.

In our study, we used homogenous and validated tests concerning sexual domains.

Our results regarding sexual functions are in accordance with those of Traa et al. (17), where no changes were found before and after colorectal cancer surgery.

Interestingly, we found a significant decrease in women’s sexual function (FSFI) as well as their satisfaction within their couple (Locke–Wallace) until 12 months after surgery. In contrast, they showed a steady sexual function (GRISS) within their couple until 12 months after surgery.

Regarding men, the evolution over time for both their own sexual function and within the couple but also their satisfaction within the couple seems stable.

Thus, these results and differences seem to point out the necessity to conduct a deeper analysis with different methodologies to assess the accuracy and veracity of these answers. Indeed, it is well-known that there are gender-related differences toward sexuality function and sexual health (18). Moreover, sexuality is a topic of privacy and in some ways may be difficult to speak about, needing trust and confidentiality (19). Even in the healthcare system, the subject brings caution and sometimes has not even been considered in the discussion or assessed before surgery between the surgeon and the patient according to a recent survey (20).

This study did not have the ambition to focus and assess specific sexual dysfunctions or sexual unwell-being after specific pathologies or subgroup of women or men. It is more an overview about the topic of sexual function and health of patients undergoing colorectal surgery.

### Strengths

The major interest of our study, contrary to those of the current literature, is to analyze sexual dysfunctions after colorectal surgery whatever indication, localization, or gender. Contrary to other studies, our analysis was based on validated questionnaires.

### Limitations

The main limit of our work is the presence of a small number of persons especially in the subgroup of women where the number of 20 that was expected was not reached. This can be explained by the need of time for patients to fill out the questionnaires because they are detailed. Thus, some patients had the tests without having completed them, whereas others were total non-responders. Moreover, a longer follow-up would have been more suitable. Other limits can be pointed out and will have to be taken into account for the next research. More recent data after 2016 have to be studied and compared with these ones; all the colorectal procedures have been included in our work, which can be a reason for bias as the number of rectal cases is less and it is well documented that sexual function is mostly affected by rectal dissection. Nevertheless, it is important to highlight that there are many studies which deal with quality of life after rectal surgeries, but there are very limited studies which deal exclusively with sexual functions after such surgeries, making our study an important one. Our study covers an extremely wide group of patients (malignant–benign, colon–rectal resections, presence–absence of stoma). The fact that it was conducted in a small group precludes any subgroup analysis. Furthermore, we can underline that studies involving larger and different populations may be interesting and valuable: thus, in future studies, we will include homosexual and bisexual patients and those without an apparent “sexuality.”

## Conclusion

Interestingly, in this monocentric study, colorectal surgery does not influence sexual function and sexual quality of life in both men and women whatever the indication until 1 year after surgery.

However, preservation of the sexual function as well as the marital satisfaction of colorectal patients should be of major concern for the involved caregivers, alongside outcomes like morbidity, mortality, or oncological results. Sexual disorders should be assessed, as other aspects of quality of life, before and after surgery to identify their occurrence and offer appropriate care. The use of standardized and validated questionnaires, if possible by involving the partner, ensures quality follow-up.

These results should be confirmed by larger multicentric studies.

## Data availability statement

The raw data supporting the conclusions of this article will be made available by the authors, without undue reservation.

## Ethics statement

This study was approved by the Health Research Ethics Board at the University of Geneva (CER 14-111). The patients/participants provided their written informed consent to participate in this study.

## Author contributions

EL, SdS, JK, NB, and FR conceived and designed the study. NC, EL, SdS, JK, and DG acquired the data. NC and EL analyzed the data. NC and EL interpreted the data. NC, EL, SdS, JK, IP, DG, NB, and FR contributed to the writing of the manuscript and to its critical revision. NC, EL, SdS, JK, IP, DG, NB, and FR approved the final version of the manuscript. All authors contributed to the article and approved the submitted version.

## References

[1] Bardakcioglu O. Advanced techniques in minimally invasive and robotic colorectal surgery. United States: Springer US (2015). Available at: https://www.springer.com/gp/book/9781489978318.

[2] Falzone L Salomone S Libra M. Evolution of cancer pharmacological treatments at the turn of the third millennium. Front Pharmacol (2018) 9:1300/full. doi: 10.3389/fphar.2018.01300/full 30483135 PMC6243123

[3] Ng SC Shi HY Hamidi N Underwood FE Tang W Benchimol EI. Worldwide incidence and prevalence of inflammatory bowel disease in the 21st century: A systematic review of population-based studies. Lancet Lond Engl (2017) 390(10114):2769–78. doi: 10.1016/S0140-6736(17)32448-0 29050646

[4] Mak WY Zhao M Ng SC Burisch J. The epidemiology of inflammatory bowel disease: East meets west. J Gastroenterol Hepatol (2020) 35(3):380–9. doi: 10.1111/jgh.14872 31596960

[5] Weledji E Ngounou E. The-anatomical-basis-for-autonomic-dysfunction-in-pelvic-surgery. Gen Surg (2020) 14:4.

[6] da Silva GM Hull T Roberts PL Ruiz DE Wexner SD Weiss EG. The effect of colorectal surgery in female sexual function, body image, self-esteem and general health: a prospective study. Ann Surg (2008) 248(2):266–72. doi: 10.1097/SLA.0b013e3181820cf4 18650637

[7] Basson R Schultz WW. Sexual sequelae of general medical disorders. Lancet Lond Engl (2007) 369(9559):409–24. doi: 10.1016/S0140-6736(07)60197-4 17276781

[8] Rosen RC Riley A Wagner G Osterloh IH Kirkpatrick J Mishra A. The international index of erectile function (IIEF): A multidimensional scale for assessment of erectile dysfunction. Urology (1997) 49(6):822–30. doi: 10.1016/S0090-4295(97)00238-0 9187685

[9] Rosen R Brown C Heiman J Leiblum S Meston C Shabsigh R. The female sexual function index (FSFI): A multidimensional self-report instrument for the assessment of female sexual function. J Sex Marital Ther (2000) 26(2):191–208. doi: 10.1080/009262300278597 10782451

[10] Locke HJ Wallace KM. Short marital-adjustment and prediction tests: Their reliability and validity. Marriage Fam Living (1959) 21:251–5. doi: 10.2307/348022

[11] Wang G Wang Z Jiang Z Liu J Zhao J Li J. Male Urinary and sexual function after robotic pelvic autonomic nerve-preserving surgery for rectal cancer. Int J Med Robot (2017) 13(1):e1725. doi: 10.1002/rcs.1725 26748601

[12] Adam J-P Denost Q Capdepont M van Geluwe B Rullier E. Prospective and longitudinal study of urogenital dysfunction after proctectomy for rectal cancer. Dis Colon Rectum (2016) 59(9):822–30. doi: 10.1097/DCR.0000000000000652 27505110

[13] Kim HJ Choi G-S Park JS Park SY Yang CS Lee HJ. The impact of robotic surgery on quality of life, urinary and sexual function following total mesorectal excision for rectal cancer: A propensity score-matched analysis with laparoscopic surgery. Colorectal Dis Off J Assoc Coloproctol G B Irel (2018) 20(5):O103–13. doi: 10.1111/codi.14051 29460997

[14] Morelli L Di Franco G Guadagni S Rossi L Palmeri M Furbetta N. Robot-assisted total mesorectal excision for rectal cancer: Case-matched comparison of short-term surgical and functional outcomes between the da Vinci xi and Si. Surg Endosc (2018) 32(2):589–600. doi: 10.1007/s00464-017-5708-5 28733738

[15] Sung H Ferlay J Siegel RL Laversanne M Soerjomataram I Jemal A. Global cancer statistics 2020: GLOBOCAN estimates of incidence and mortality worldwide for 36 cancers in 185 countries. CA Cancer J Clin (2021) 71(3):209–49. doi: 10.3322/caac.21660 33538338

[16] Wong MCS Huang J Lok V Wang J Fung F Ding H. Differences in incidence and mortality trends of colorectal cancer worldwide based on sex, age, and anatomic location. Clin Gastroenterol Hepatol Off Clin Pract J Am Gastroenterol Assoc (2021) 19(5):955–966.e61. doi: 10.1016/j.cgh.2020.02.026 32088300

[17] Traa MJ Roukema JA De Vries J Rutten HJT Langenhoff B Jansen W. Biopsychosocial predictors of sexual function and quality of sexual life: a study among patients with colorectal cancer. Transl Androl Urol (2015) 4(2):206–17. doi: 10.3978/j.issn.2223-4683.2015.03.01 PMC4708118 26816825

[18] Mollaioli D Ciocca G Limoncin E Di Sante S Gravina GL Carosa E. Lifestyles and sexuality in men and women: The gender perspective in sexual medicine. Reprod Biol Endocrinol RBE (2020) 18:10. doi: 10.1186/s12958-019-0557-9 PMC7025405 32066450

[19] Hinchliff S Fileborn B Alba B Lyons A Minichiello V Barrett C. Talking about sex with friends: perspectives of older adults from the sex, age & me study in Australia. Cult Health Sex (2021) 23(3):367–82. doi: 10.1080/13691058.2019.1710568 32609066

[20] Dames NB Squire SE Devlin AB Fish R Bisset CN Tozer P. ‘Let’s talk about sex’: A patient-led survey on sexual function after colorectal and pelvic floor surgery. Colorectal Dis (2021) 23(6):1524–51. doi: 10.1111/codi.15598 PMC9291989 33615666

